# PF-429242, a Subtilisin Inhibitor, Is Effective *in vitro* Against *Leishmania infantum*

**DOI:** 10.3389/fmicb.2021.583834

**Published:** 2021-01-28

**Authors:** Patrícia de Almeida Machado, Pollyanna Stephanie Gomes, Victor Midlej, Elaine Soares Coimbra, Herbert Leonel de Matos Guedes

**Affiliations:** ^1^Laboratório Interdisciplinar de Pesquisas Médicas, Instituto Oswaldo Cruz, Fundação Oswaldo Cruz (Fiocruz), Rio de Janeiro, Brazil; ^2^Laboratório de Imunofarmacologia, Instituto de Biofísica Carlos Chagas Filho, Universidade Federal do Rio de Janeiro (UFRJ), Rio de Janeiro, Brazil; ^3^Núcleo de Pesquisa em Parasitologia (NUPEP), Instituto de Ciências Biológicas, Universidade Federal de Juiz de Fora, Juiz de Fora, Brazil; ^4^Laboratório de Ultraestrutura Celular, Instituto Oswaldo Cruz, Fundação Oswaldo Cruz (Fiocruz), Rio de Janeiro, Brazil; ^5^UFRJ Campus Duque de Caxias Professor Geraldo Cidade – Universidade Federal do Rio de Janeiro, Duque de Caxias, Brazil; ^6^Instituto de Microbiologia Paulo de Góes, Universidade Federal do Rio de Janeiro (UFRJ), Rio de Janeiro, Brazil

**Keywords:** visceral leishmaniasis, leishmaniasis chemotherapy, serine protease, subtilisin, PF-429242

## Abstract

PF-429242 is an inhibitor of subtilisin, an important protease found in *Leishmania*. However, studies regarding the effect of PF-429242 on *Leishmania* are scarce. In this work we evaluated the antileishmanial effect of PF-429242 against *Leishmania infantum* and the mechanism involved in the death of the parasite. PF-429242 had low toxicity against mammalian cells (peritoneal macrophages) (CC_50_ = 189.07 μM) and presented activity against *L. infantum* promastigotes (IC_50_ = 2.78 μM) and intracellular amastigotes (IC_50_ = 14.07 μM), indicating selectivity toward the parasite. Transmission electron microscopy (TEM), as well as staining of *L. infantum* promastigotes with MitoTracker^®^ Red, rhodamine 123 and MitoSOX, revealed that the mitochondria was a potential target of PF-429242. In addition, PF-429242 caused an accumulation of neutral lipids in promastigotes, which was demonstrated by Nile Red staining and TEM, and induced oxidative stress (H_2_DCFDA staining). Furthermore the formation of autophagic vacuoles in *L. infantum* promastigotes was observed by MDC staining and TEM. However, the killing induced by PF-429242 in *L. infantum* promastigotes appeared to be unrelated to apoptosis and/or necrosis as there was no phosphatidylserine externalization, DNA fragmentation or alterations in the permeability of the parasite plasma membrane, as assessed by annexin V-FITC, TUNEL and propidium iodide staining, respectively. The morphological and ultrastructural evaluation of the promastigotes by optical microscopy, scanning electron microscopy (SEM) and TEM, revealed the presence of parasites with flagellar defects. TEM analysis of the intracellular amastigotes indicated that mitochondrial damage and autophagy could also be involved in the death of these forms after treatment with PF-429242. In addition, PF-429242 treatment stimulated NO production from infected macrophage, but only at a high concentration (100 μM), as well as an increase of TNF levels after treatment with 10 μM of PF-429242. The compound did not stimulate ROS or IL-10 production. Together, these data highlight the antileishmanial potential of PF-429242, inducing several cellular alterations in the parasite, such as mitochondrial damage, neutral lipids accumulation, oxidative stress and autophagy which culminate in the death of *L. infantum*, as well as modulating host cellular responses that favor the development of an immune response against the parasite.

## Introduction

Visceral leishmaniasis (VL) is a neglected tropical disease caused by parasites of the *Leishmania* genus (Gidey et al., 2019). VL is the most severe of all forms of human leishmaniasis, causing a systemic disease that in most cases is fatal if not treated correctly (Burza et al., 2018). The symptoms include weight loss, fever, splenomegaly, hepatomegaly and anemia (Steverding, 2017). Around the world an estimated 300,000 new cases and over 20,000 deaths occur annually (WHO, 2019). VL is caused by *Leishmania donovani* in Asia and Africa, and by *Leishmania infantum* in Central Asia, the Mediterranean Basin, Middle East and Latin America (Burza et al., 2018).

Chemotherapy of VL relies on the use of treatments that have severe side effects, including pentavalent antimonials (Glucantime^®^ or Pentostam^®^), amphotericin B and its lipid formulations, miltefosine and paromomycin (Sundar, 2015; van Griensven and Diro, 2019). However, all these drugs have limited use, because of their high cost, toxicity, the requirement of long-term treatment and the emergence of parasite resistance (Ghorbani and Farhoudi, 2018). Therefore, the search for new treatment alternatives for VL is urgent and extremely necessary.

Proteases are proteolytic enzymes that hydrolyze peptide bonds present in proteins or peptides, giving rise to small peptides and/or amino acids (Turk, 2006). Serine proteases are an important group within the proteases and are involved in the pathogenesis of several parasitic diseases, such as schistosomiasis and onchocerciasis (Sakanari et al., 1989). In *Leishmania* parasites, serine proteases are involved in infectivity, differentiation of promastigotes into amastigotes, proliferation, virulence and protection against oxidative stress (Machado et al., 2019).

Subtilisin belongs to the SB clan and S8 family, which is the second largest family of serine proteases (Rawlings and Barrett, 1994). Swenerton et al. (2010) identified and phenotypically characterized the *Leishmania* subtilisin. These authors reported that in *L. major* only one allele in the subtilisin gene could be silenced, because the deletion of the two alleles is lethal to the parasite. In contrast, for *L. donovani* the silencing of the two alleles is not lethal, however, these parasites exhibit several abnormalities: difficulty in the differentiation from promastigotes to amastigotes, increased susceptibility to oxidative stress, reduced virulence in mice and hamsters, binucleated amastigotes with the flagellum outside the expected location and with altered protein levels in relation to the wild-type parasites (Swenerton et al., 2010). These facts highlight the importance of subtilisin for *Leishmania* parasites, suggesting that this serine protease could be a therapeutic target for VL.

**FIGURE 1:**
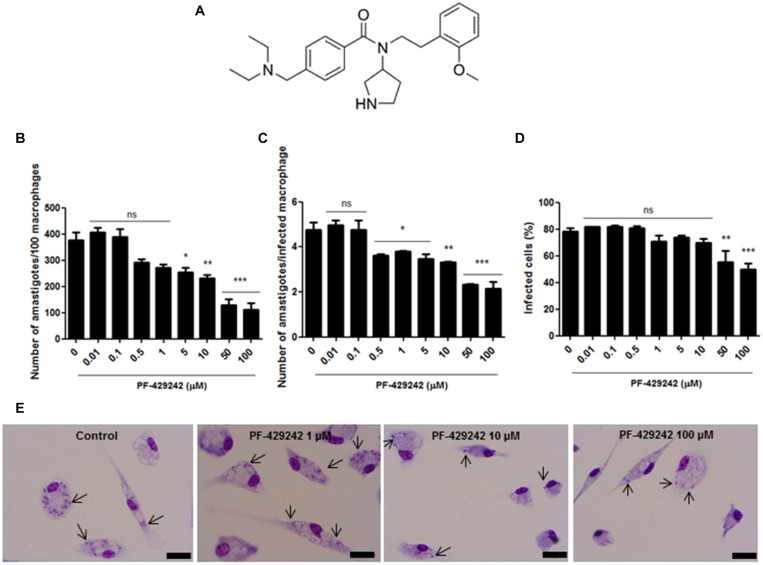
Chemical structure of PF-429242 and effect of this compound against intracellular amastigotes of *L. infantum*. **(A)** Chemical structure of PF-429242. **(B–E)** Peritoneal macrophages from BALB/c mice were infected with *L. infantum* and treated with several concentrations of PF-429242 (100, 50, 10, 5, 1, 0.5, 0.1, and 0.01 μM) for 72 h at 37°C and 5% CO_2_. The cells were fixed with ethanol, stained with Giemsa and counted. A total of 100 macrophages were assessed and the total number of amastigotes **(B)**, the number of amastigotes per infected macrophage **(C)** and the percentage of infected macrophages **(D)** were determined. Bars indicate the mean ± SEM. Statistical analyses were made using One-way ANOVA followed by Tukey post-test to compare the untreated control group with the other treatments: ***p < 0.001, **p < 0.01, *p < 0.05 and ns (not significant). **(E)** Photomicrographs of the control group (untreated) and those treated with PF-429242 (1, 10, and 100 μM). Bar = 10 μm. Arrows indicate the amastigotes inside macrophages.

Aminopyrrolidineamide compounds are *N*-(3-pyrrolidinyl) benzamide derivatives. *N*-(3-pyrrolidinyl)benzamides have already been patented with high selectivity and affinity by D_3_ and/or D_4_ receptors (dopamine receptors) and weak action on the D_2_ receptor, exerting psychotropic actions in some diseases such as emotional and mental disorders, anxiety, sleep disorders, schizophrenia, drug abuse and personality disorders. In addition, these compounds can exert central and/or peripheral actions in Parkinson’s disease and sexual disorders (Ohmori et al., 1997). PF-429242 (Figure 1A), an aminopyrrolidineamide (Hyrina et al., 2017), was initially developed as a hypolipidemic agent by high throughput screening of Pfizer compounds (Uchida et al., 2016). This compound is an inhibitor of the protease subtilisin kexin isozyme-1 (SKI-1), an important regulator of lipid homeostasis and consequently of the sterol regulatory element-binding protein (SREBP) pathway (Hyrina et al., 2017). PF-429242 has been shown to have anti-viral activity, as Pasquato et al. (2012) demonstrated against arenaviruses. In addition, it has been shown that PF-429242 impairs the onset of hepatitis C virus infection by inhibiting the entry of this pathogen into the host cell (Blanchet et al., 2015). However, there are no studies showing the effect of this compound on *Leishmania* parasites. Therefore, this work evaluated, for the first time, the antileishmanial effect of PF-429242 and the mechanism involved in the death of *L. infantum* treated with this compound.

## Materials and Methods

### Parasites

*Leishmania (Leishmania) infantum* (MHOM/MA/67/ITMAP-263), originally isolated from humans in Morocco, North Africa, was kindly provided by Dr. Eduardo Caio Torres-Santos. Promastigotes were grown at 25°C in BHI medium (HiMedia) plus hemin, folic acid, 0.1% penicillin and streptomycin solution (Sigma-Aldrich) and 10% fetal bovine serum (FBS) (Cultilab), pH 7.2 – 7.4. This culture of promastigotes has a logarithmic phase between days 1 and 3 and the stationary phase begins on day 4, for this reason, tests on promastigotes were carried out using cultures on days 2 or 3 of growth, while macrophage infections were always performed with the cultures on day 4 of growth. From day 5, this culture was discarded. In addition, promastigotes were used only until the seventh passage after isolation from animals.

### Compounds

The compound tested in this work, PF-429242, was purchased from Sigma-Aldrich and diluted in deionized water. Amphotericin B, obtained from Cristália, was used as a reference drug in antileishmanial tests against promastigote and amastigote forms, it was also diluted with water and the following concentrations were used in the tests: 0.5, 0.25, 0.12, 0.06, 0.03, and 0.01 μM. Antimycin A was purchased from Sigma-Aldrich and used at 10 μM. Miltefosine was obtained from Cayman Chemical and used at 8 μM.

### Mice

Female BALB/c mice were obtained from the Central Animal Facility of Universidade Federal do Rio de Janeiro (UFRJ), Fundação Oswaldo Cruz-Fiocruz and Universidade Federal de Juiz de Fora (UFJF). All procedures for use and maintenance were performed according to protocols approved by the Ethical Committee for Animal Handling (CEUA 080/2018 from UFRJ, L-009/2020 from Fiocruz and 008/2018 from UFJF). The animals were used at 5–10 weeks old and with a weight of 20–25 g. The mice were kept under controlled light/dark cycle conditions and temperature (22°C), with access to water and food *ad libitum*.

### Anti-promastigote Activity

Promastigote forms in the log phase of growth were distributed in 96-well plates at a concentration of 3 × 10^6^ cells/well in RPMI medium without phenol red (HiMedia) supplemented with 10% FBS and 0.1% penicillin and streptomycin solution. PF-429242 was added at concentrations of 100, 50, 10, 5, 1, 0.5, 0.1, and 0.01 μM. The tests were performed in duplicate and *L. infantum* promastigotes maintained in the culture medium was used as a control. After a 72 h period of incubation at 25°C, MTT (Sigma-Aldrich) was added and the plates were incubated for 4 h. The reaction was stopped by the addition of a 0.7% isopropanol/HCl solution and read on a spectrophotometer (Multiskan EX) at 570 nm. Results were calculated as percentage inhibition of promastigote growth compared to the control. IC_50_ values were determined using the GraFit5 program. Three independent experiments were performed.

### Anti-amastigote Activity

Peritoneal macrophages from BALB/c mice, obtained by peritoneal lavage, as previously described by Bisgaard et al. (2016). Macrophages were added to 13 mm glass coverslips in 24-well plates at a concentration of 2 × 10^6^ cells/mL in RPMI-1640 medium (Cultilab) containing 10% FBS and 0.5% penicillin and streptomycin solution. The plates were incubated at 37°C with 5% CO_2_ for 1 h. After this time, the plates were washed with PBS and again incubated in RPMI-1640 with 10% FBS and 0.5% penicillin and streptomycin solution at 37°C with 5% CO_2_. The following day, the cells were washed with PBS and infected with *L. infantum* promastigotes in the stationary phase of growth (10:1 ratio) for 24 h. The wells were then washed with PBS to remove extracellular parasites and PF-429242 was added at different concentrations (100, 50, 10, 5, 1, 0.5, 0.1, and 0.01 μM) in RPMI-1640 medium containing 4% horse serum (Laborclin) and 0.5% penicillin and streptomycin solution. The tests were performed in duplicate and infected macrophages with *L. infantum* maintained in the culture medium were used as a control. After 72 h of treatment at 37°C and 5% CO_2_, the coverslips were removed, the cells fixed with ethanol, stained with Giemsa and dehydrated in acetone/xylol. The coverslips were placed on slides and for each coverslip a total of 100 macrophages were assessed for the count of intracellular parasites in terms of the total number of amastigotes, the number of amastigotes per infected macrophage and the percentage of infected macrophages. Results were calculated as percentage inhibition of amastigote growth compared to the control and IC_50_ values were determined using the GraFit5 program. Three independent experiments were performed. In addition, the slides were analyzed on an optical microscope (Olympus BX53), with 1,000× magnification and photomicrographs were taken using an Olympus DP73 camera.

### Cytotoxicity Assay

Peritoneal macrophages from BALB/c mice, obtained by peritoneal lavage, were distributed in 96-well plates at a concentration of 2 × 10^6^ cells/mL in RPMI-1640 medium containing 10% FBS and 0.5% penicillin and streptomycin solution. The plates were incubated at 37°C with 5% CO_2_. The following day, the cells were washed with PBS and PF-429242 was added at different concentrations (200, 100, 50, 25, 12.5, and 6.25 μM). After a 72 h period of incubation at 37°C and 5% CO_2_, MTT was added and the plates were incubated again for 2 h. The reaction was stopped by the addition of a 0.7% isopropanol/HCl solution and read on a spectrophotometer at 570 nm. Results were calculated as percentage of cell death compared to the untreated control. CC_50_ values were determined using the GraFit5 program. Three independent experiments were realized.

### *In vitro* Treatment With PF-429242

To evaluate the mode of action of PF-429242 in *L. infantum*, log phase promastigotes were counted in a Neubauer chamber and the concentration was adjusted to 5 × 10^6^ parasites/mL in 5 mL RPMI medium without phenol red supplemented with 10% FBS and 0.1% penicillin and streptomycin solution. PF-429242 was added at two concentrations (3 and 6 μM) and incubated for 72 h at 25°C. These concentrations correspond to one and two-fold the IC_50_ of this protease inhibitor (approximated values). After incubation with PF-429242 the parasites were washed in PBS and the parasite concentrations were adjusted for the subsequent stainings described below.

#### Determination of the Mitochondrial Membrane Potential (ΔΨm)

The ΔΨm of *L. infantum* promastigotes treated with PF-429242 (3 and 6 μM) was evaluated using two distinct fluorescent probes: MitoTracker^®^ Red CM-H2XROS (Life Technologies) and rhodamine 123 (Sigma-Aldrich). These probes accumulate in mitochondria and this accumulation is dependent on the ΔΨm (Chazotte, 2011). For staining with MitoTracker^®^ Red, the parasite concentration was adjusted to 5 × 10^6^ promastigotes/mL in 1 mL PBS, MitoTracker was added at a final concentration of 500 nM and incubated for 40 min at 25°C in the dark. After incubation, the cells were washed with PBS and read in a spectrofluorimeter (FLx800) at 540/600 nm of excitation/emission, respectively. For staining with rhodamine 123, the concentration was adjusted to 1 × 10^7^ promastigotes/mL in 200 μL PBS, rhodamine 123 was added at a final concentration of 0.5 μg/mL and incubated for 20 min at 25°C in the dark. The reading was done in a spectrofluorimeter at 485/528 nm of excitation/emission, respectively. In both cases, *L. infantum* promastigotes maintained in the culture medium were used as a negative control and promastigotes treated with carbonyl cyanide-4-(trifluoromethoxy) phenylhydrazone (FCCP) at 1 μM was used as a positive control. Three independent experiments were performed.

#### Measurement of Mitochondrial Superoxide Production

MitoSOX (Invitrogen) is a probe that is selectively targeted to mitochondria in live cells and is oxidized by superoxide radical. After treatment with PF-429242 (3 and 6 μM), the parasite concentration was adjusted to 2 × 10^7^ promastigotes/mL in 200 μL PBS and MitoSOX was added at a final concentration of 5 μM and used according to manufacturer’s instructions. Promastigotes were incubated for 20 min and then read in a spectrofluorimeter at 540/600 nm of excitation/emission, respectively. *L. infantum* promastigotes maintained in culture medium were used as a negative control and cells treated with antimycin A (10 μM) were used as a positive control. Three independent experiments were performed.

#### Evaluation of Neutral Lipid Accumulation

Neutral lipid accumulation in treated *L. infantum* promastigotes was determined by labeling with Nile Red, a lipophilic substance capable of binding to neutral intracellular lipids. There is a linear correlation between its fluorescence and neutral lipid content within cells (Alemán-Nava et al., 2016). Briefly, after treatment with PF-429242 (3 and 6 μM), the concentration of *L. infantum* parasites was adjusted to 1 × 10^7^ promastigotes/mL in 200 μL PBS, then incubated with Nile Red at a final concentration of 10 μg/mL for 30 min at 25°C in the dark. Fluorescence intensity was evaluated in a spectrofluorimeter at 485/528 nm of excitation/emission, respectively. Miltefosine (8 μM) was used as a positive control and promastigotes maintained in culture medium were used as a negative control. Three independent experiments were performed.

#### Measurement of Intracellular Reactive Oxygen Species (ROS) Levels

ROS levels were determined using 2′,7′-dichlorodihydrofluorescein diacetate (H_2_DCFDA) (Invitrogen), a non-fluorescent substance, but when oxidized in the presence of ROS becomes dichlorofluorescein (DCF), a highly fluorescent compound (Sardar et al., 2013). After treatment with PF-429242 (3 and 6 μM), parasite concentration was adjusted to 2 × 10^7^ promastigotes/mL in 200 μL PBS. The probe was added at final concentration of 20 μM and incubated for 30 min. The reading was performed in a spectrofluorimeter at 485/528 nm of excitation/emission, respectively. H_2_O_2_ (0.5 μM) was used as a positive control and promastigotes maintained in culture medium were used as a negative control. Three independent experiments were done.

#### Evaluation of Plasma Membrane Permeability

The evaluation of plasma membrane permeability was realized using propidium iodide (PI) staining (Sigma-Aldrich). This probe is a fluorescent substance that labels the DNA of cells with damaged plasma membrane, since under these conditions it has the ability to penetrate the cell (Masango et al., 2015). After treatment with PF-429242 (3 and 6 μM), the parasite concentration was adjusted to 1 × 10^7^ cells/mL in 200 μL PBS. Cells were labeled with PI at a final concentration of 1 μg/mL, and incubated for 15 min. Assessment of fluorescence intensity was achieved in a spectrofluorimeter at 540/600 nm of excitation/emission, respectively. Promastigotes incubated at 65°C for 15 min in a water bath were used as a positive control and promastigotes maintained in the culture medium were used as a negative control. Three independent experiments were done.

#### Evaluation of Phosphatidylserine (PS) Externalization

Externalization of PS is a marker of apoptosis. After an apoptotic stimulus, PS normally found in the inner leaflet of the plasma membrane, translocates to the outer leaflet of the membrane (Sen et al., 2007). PS is a phospholipid present in the plasma membrane of *Leishmania* parasites (Tripathi and Gupta, 2003) and the externalization of PS residues in apoptotic cells can be detected by annexin V staining (Taruvai Kalyana Kumar et al., 2016). After treatment with PF-429242 (3 and 6 μM), the cell concentration was adjusted to 1 × 10^7^ cells/mL in 200 μL calcium buffer, 1 μl annexin V-FITC (Invitrogen) was added to each of the samples then incubated for 30 min. The samples were read in a spectrofluorimeter at 485/528 nm (excitation/emission). Treatment with miltefosine (8 μM) was used as a positive control and promastigotes maintained in culture medium were used as a negative control. Three independent experiments were done. In parallel, PI staining was carried out to control for the integrity of the plasma membrane of the cells and to avoid staining of PS on the inner leaflet of the plasma membrane.

#### Evaluation of DNA Fragmentation

DNA fragmentation of *L. infantum* promastigotes treated with PF-429242 was determined by TUNEL (TdT-mediated dUTP-X nick end labeling) technique, using a commercial kit (*In Situ* Cell Death Detection Kit, Fluorescein - Roche). This technique consists of the catalytic incorporation of the nucleotide, dUTP, coupled to fluorescein, at strand breaks of the DNA, through the terminal transferase enzyme (taken from the manufacturer’s instructions). Promastigotes treated or not with PF-429242 (3 and 6 μM) were washed with PBS and the cell concentration was adjusted to 3 × 10^7^ promastigotes/mL in 1 mL PBS. The cells were fixed with 4% paraformaldehyde for 1 h and permeabilized with 0.1% Triton X-100 in 0.1% sodium citrate for 2 min. Promastigotes were stained with TUNEL according to the manufacturer’s instructions and fluorescence intensity was determined using a spectrofluorimeter at 485/528 nm (excitation/emission). Promastigotes treated with 5 μg/mL DNase for 10 min were used as a positive control and promastigotes maintained in culture medium were used as a negative control. Three independent experiments were performed.

#### Evaluation of Autophagic Vacuoles Formation

The labeling of autophagic vacuoles was realized using monodansylcadaverine (MDC), an autofluorescent substance that labels autophagic vacuoles (Bera et al., 2003). After treatment with PF-429242 (3 and 6 μM), the parasite concentration was adjusted to 1 × 10^7^ promastigotes/mL in 300 μl PBS and incubated for 60 min at 25°C with MDC (Sigma-Aldrich) at a final concentration of 100 μM. Next, the parasites were washed with PBS, fixed with 4% paraformaldehyde and fluorescence intensity was determined using a spectrofluorimeter at 335/518 nm (excitation/emission). Parasites growing for 3 days under malnutrition conditions (in PBS) were used as a positive control and promastigotes maintained in culture medium was used as a negative control. Three independent experiments were performed.

#### Determination of the Morphology by Optical Microscopy

The morphology of *L. infantum* promastigotes, treated with PF-429242 (3 and 6 μM), was determined after washing with PBS, fixation with 4% paraformaldehyde and labeling with Giemsa. Posteriorly, the slides were mounted and photographed on an optical microscope with 1,000× magnification (Olympus BX53), using an Olympus DP73 camera. Quantitative analysis was performed on the photomicrographs, in which 200 promastigotes were counted in each group (control, PF-429242 3 μM and PF-429242 6 μM), differentiating parasites without flagellar defects (healthy) and with flagellar defects (defective). Quantitative analysis of each type of flagellar defect were also performed. Three independent experiments were performed.

#### Scanning Electron Microscopy (SEM) of *L. infantum* Promastigotes

*Leishmania infantum* promastigotes treated with PF-429242 (3 and 6 μM) were spread on coverslips pre-treated with 0.01% poly-L-lysine (Sigma-Aldrich) and fixed in 2.5% glutaraldehyde (Sigma-Aldrich) in 0.1 M sodium cacodylate buffer at room temperature for 1 h. The parasites were post-fixed in 1% osmium tetroxide (OsO_4_) for 15 min and then dehydrated in an ascending series of ethanol – 7.5, 15, 30, 50, 70, 90, and 100% (v/v) – for 15 min at each step. Next, the samples were critical point dried with CO_2_, sputter-coated with a 15 nm thick layer of gold and observed in a Jeol JSM 6390 (Tokyo, Japan) scanning electron microscope. Promastigotes maintained in the culture medium were used as a negative control. Two independent experiments were performed.

### Transmission Electron Microscopy (TEM) of *L. infantum* Promastigotes and Amastigotes

After treatment of *L. infantum* promastigotes (3 and 6 μM) and peritoneal macrophages infected with *L. infantum* amastigotes (14 and 28 μM) with PF-429242, the cells were fixed in 2.5% glutaraldehyde in 0.1 M sodium cacodylate buffer (room temperature for 1 h) and post-fixed in 1% osmium tetroxide and 0.8% potassium ferrocyanide solution. An increasing concentration series of acetone (70, 90, and 100%) were used to dehydrate the cells, which were after embedded in Epon resin and polymerized at 60°C. Ultrathin sections (60–70 nm thick) were stained with 5% uranyl acetate and lead citrate and examined using a Jeol JEM 1011 (Tokyo, Japan) transmission electron microscope. Cells maintained in the culture medium were used as a negative control. Two independent experiments were performed.

### Measurement of Nitric Oxide (NO) Levels Produced by *L. infantum*-Infected Macrophages

During the anti-amastigote assay, supernatant from cultures of *L. infantum-*infected macrophages, treated or not with PF-429242 (100, 10, 1, 0.1, and 0.01 μM), was collected and kept frozen until analysis. NO levels were determined in 50 μL aliquots to which 50 μL of Griess reagent (1% sulfanilamide in 2.5% of H_3_PO_4_ + 0.1% *N*-1-diidrocloride naftiletilenodiamine in 2.5% H_3_PO_4_) was added in a 96-well microplate (Green et al., 1982). Nitrite content was quantified by comparison with a sodium nitrite standard curve. The absorbance was measured at 540 nm using a spectrophotometer (Multiskan MS). The assays were carried out in duplicate and three independent experiments were realized. IFN-γ (obtained from L1210 cell supernatant) at 1 ng/mL was used as a positive control.

### Measurement of Oxygen Reactive Species (ROS) Levels in *L. infantum*-Infected Macrophages

Macrophages from BALB/c mice, obtained by peritoneal lavage, were plated in 96-well microplates (1 × 10^6^ cells/mL) and infected with *L. infantum* promastigotes (10:1 ratio) for 24 h at 37°C. Non-adherent promastigotes were washed; PF-429242 (100, 10, 1, 0.1, and 0.01 μM) was added and maintained for 72 h. After this time, plates were washed with PBS and H_2_DCFDA (20 μM) was added for 30 min. The read was made in a spectrofluorometer (485/528 nm). *Leishmania amazonensis*-infected macrophages maintained in the culture medium were used as a negative control and those stimulated with H_2_O_2_ (4 mM) for 30 min were used as a positive control. Three independent experiments were performed.

### Measurement of TNF and IL-10 Levels Produced by *L. infantum*-Infected Macrophages

To evaluate the TNF and IL-10 levels, the supernatant from the culture of macrophages infected with *L. infantum* amastigotes and treated with PF-429242 (100, 10, 1, 0.1, and 0.01 μM) for 72 h was used. The TNF and IL-10 levels were detected by sandwich enzyme-linked immunosorbent assay (ELISA) ABTS protocol using the diagnostic kits obtained from Peprotech and the protocol was developed according to the manufacturer’s instructions. The absorbance was read on a spectrophotometer using 405 nm with wavelength correction set at 650 nm. Concentrations of TNF and IL-10 were calculated from the standard curve for each cytokine. Supernatant from cells maintained only in culture medium was used as a negative control for both cytokines. Supernatant from cells treated with IFN-γ (1 ng/mL) was used as a positive control for the production of TNF and treated with LPS (1 μg/mL) was used as a positive control for the production of IL-10. Three independent experiments were performed.

### Statistical Analysis

For all the tests, three independent experiments were performed. To determine the CC_50_ and IC_50_ values and for the construction of the graphs three independent experiments were performed, each in duplicate or triplicate or quadruplicate (depending on the test). The average of each independent experiment was calculated and the average of the three independent experiments was used in the statistical analysis. All graphs were generated using the GraphPad Prism 5 software and the data are presented as mean ± SEM. Statistical analysis was made using One-way ANOVA followed by Tukey post-test: *p* < 0.001 (^∗∗∗^); *p* < 0.01 (^∗∗^); *p* < 0.05 (^∗^) and ns (not significant).

## Results

### PF-429242 Induces Killing of *L. infantum* Promastigotes and Amastigotes and Has Low Toxicity Against Host Cells

First, the toxicity of the protease inhibitor, PF-429242, against mammalian cells was evaluated. For this, peritoneal macrophages from BALB/c mice were used. PF-429242 showed low toxicity against these host cells with a CC_50_ value of 189.07 μM (Table 1) and causing cell death only at the maximum concentration used (200 μM) (data not shown). The effect of this compound was also determined against the two developmental forms of *L. infantum*, the promastigotes and the intracellular amastigotes. PF-429242 showed good results against both these forms, with IC_50_ values of 2.78 ± 0.84 and 14.07 ± 0.59 μM for the promastigotes and the amastigotes, respectively (Table 1). It is important to highlight that PF-429242 was 68 times more toxic to promastigotes (selectivity index, SI = 68) and 13 times more toxic to amastigotes (SI = 13) compared to the host cells (Table 1).

**TABLE 1:** Effect of PF-429242 against peritoneal macrophages and promastigotes and intracellular amastigotes of *L. infantum*.

	Cytotoxicity against peritoneal macrophages - CC_50_ (μM)^*a*^	Antileishmanial activity against *L. infantum*			
		Promastigotes		Intracellular amastigotes	
		IC_50_ (μM)^*b*^	SI^*c*^	IC_50_ (μM)^*b*^	SI^*c*^
PF-429242	189.07 ± 12.29	2.78 ± 0.84	68	14.07 ± 0.59	13
AmB^*d*^	–	0.12 ± 0.005	–	0.05 ± 0.004	–

**^*a*^Cytotoxic concentration of 50% of macrophages (CC_50_). ^*b*^Inhibitory concentration of 50% of parasite growth (IC_50_). ^*c*^Selectivity index, calculated by: CC_50_ against macrophages/IC_50_ against parasite. ^*d*^Amphotericin B was used as reference drug*.*

At 100, 50, 10, and 5 μM, PF-429242 significantly reduced the total number of *L. infantum* intracellular amastigotes (Figure 1B); at 100, 50, 10, 5, 1, and 0.5 μM, this compound reduced the number of amastigotes per infected macrophage (Figure 1C); and at 100 and 50 μM of PF-429242, the percentage of macrophages infected with *L. infantum* amastigotes was also significantly reduced (Figure 1D). To illustrate the anti-amastigote effect of PF-429242, photomicrographs were taken of the infected macrophages treated with 100, 10, and 1 μM of this compound. Figure 1E illustrates the reduction in amastigote number after treatment with PF-429242 when compared with the untreated control, proving the effect of this compound on intracellular amastigotes of *L. infantum*.

### PF-429242 Causes Damage to the Mitochondrion of the *L. infantum* Promastigotes

To evaluate the mechanism of action of PF-429242, *L. infantum* promastigotes were treated with 3 and 6 μM of this compound. These concentrations correspond to one and two-fold the IC_50_ value of this protease inhibitor (approximated values).

**FIGURE 2:**
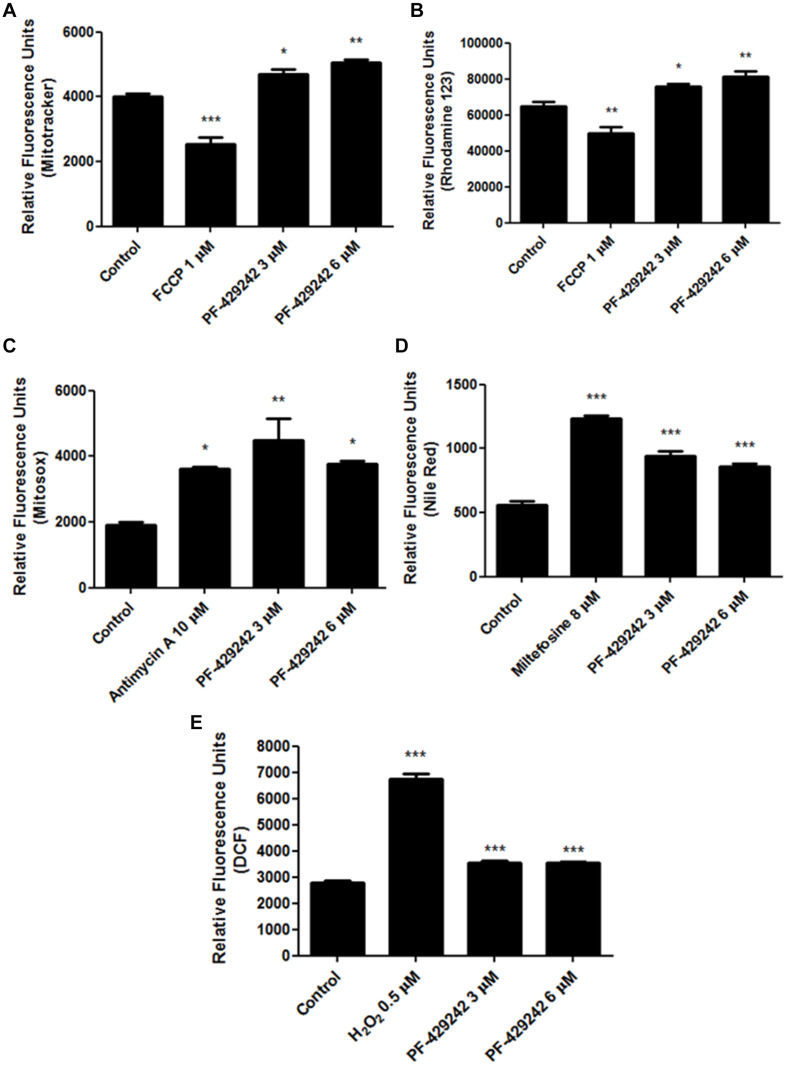
Changes in *L. infantum* promastigotes related to mitochondrial damage, neutral lipids and oxidative stress induced by treatment with PF-429242. *L. infantum* promastigotes were treated with PF-429242 (3 and 6 μM) for 72 h at 25°C. These concentrations correspond to approximated values of 1× and 2× IC_50_. Parasites were then stained with MitoTracker^®^ Red CM-H2XROS **(A)**; rhodamine 123 **(B)**; MitoSOX **(C)**; Nile Red **(D)** or H_2_DCFDA **(E)**. MitoTracker^®^ Red CM-H2XROS and rhodamine 123 were used to evaluate the ΔΨm, MitoSOX was used to detect superoxide production, Nile Red staining detected the neutral lipids accumulation and H_2_DCFDA was used to assess the ROS levels. FCCP (1 μM) **(A,B)**, antimycin A (10 μM) **(C)**, miltefosine (8 μM) **(D)**, and H_2_O_2_ (0.5 μM) **(E)** were used as a positive controls. Fluorescence intensity was evaluated in a spectrofluorometer at 485/528 nm of excitation/emission **(B,D,E)** or at 540/600 **(A,C)**. Statistical analyses were made using One-way ANOVA followed by Tukey post-test to compare the untreated control group with the other treatments: ***p < 0.001, **p < 0.01, *p < 0.05.

Treatment of *L. infantum* promastigotes with PF-429242 caused damage to the parasite mitochondrion, increasing the ΔΨ_*m*_ (hyperpolarization) when compared with the untreated control, as assessed by staining with MitoTracker Red (Figure 2A – Relative fluorescence units: ∼4,000 in control, ∼4,700 in cells treated with 3 μM PF-429242 and ∼5,000 after treatment with 6 μM PF-429242) and rhodamine 123 (Figure 2B – Relative fluorescence units: ∼65,000 in control, ∼76,000 in cells treated with 3 μM PF-429242 and ∼81,000 in treatment with 6 μM PF-429242). FCCP was used as a positive control and reduced the ΔΨ_*m*_ (despolarization) after staining with both probes (Figures 2A,B).

In addition, PF-429242 increased the mitochondrial superoxide production by approximately 135% (at 3 μM) and approximately 96% (at 6 μM), when compared with the untreated control, which was observed by staining with MitoSOX (Figure 2C). It is interesting to highlight that the PF-429242 increased the mitochondrial superoxide production similar to that of antimycin A, which was used as the positive control (Figure 2C).

Related to the mitochondrial damage, the PF-428242 treatment also caused an accumulation of neutral lipids in *L. infantum* promastigotes, when compared with untreated parasites, which was determined by staining with Nile Red (Figure 2D). The treatment with 3 μM PF-429242 increased the accumulation of neutral lipids by about 70%, and with 6 μM the increase was about 55% as compared to the untreated control. Miltefosine was used as a positive control and also led to an accumulation of neutral lipids, with an increase of about 122% (Figure 2D). Taken together, these results suggest that the mitochondrion of *L. infantum* is one the potential target of PF-429242.

### PF-429242 Induces Oxidative Stress in *L. infantum* Promastigotes

PF-429242 treatment with different concentrations induced oxidative stress in *L. infantum* promastigotes. This could be seen through an increase in ROS production after staining with H_2_DCFDA, when compared with the untreated control. The treatment with PF-429242 at 3 and 6 μM increased ROS production by about 26% (Figure 2E). As a positive control, H_2_O_2_ was used, which also increased the ROS levels by about 141% (Figure 2E).

### The Killing Induced by PF-429242 in *L. infantum* Promastigotes Seems to Be Unrelated to Apoptosis-Like and/or Necrosis

**FIGURE 3:**
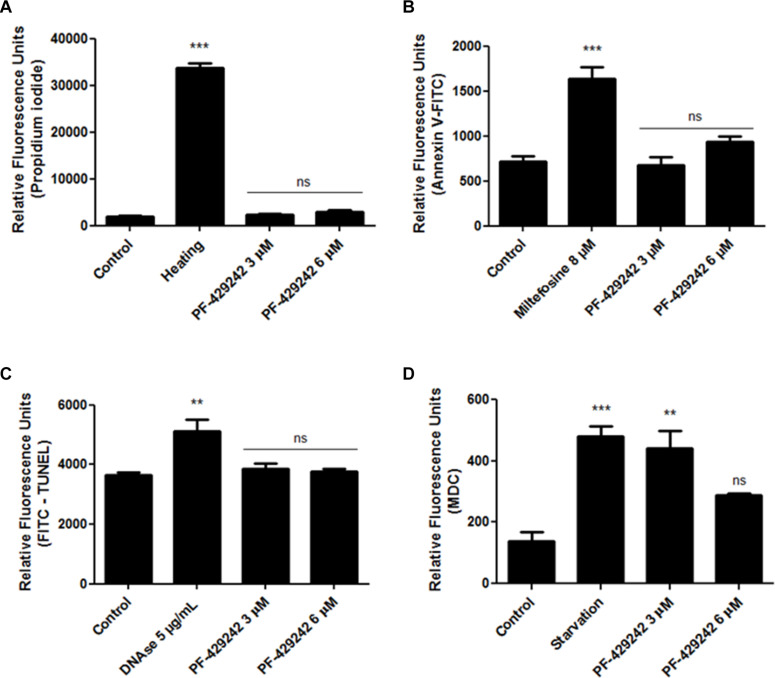
Effect of PF-429242 treatment on permeability of plasma membrane, PS externalization, DNA fragmentation and autophagic vacuoles formation. *L. infantum* promastigotes were treated with PF-429242 (3 and 6 μM) for 72 h at 25°C. Parasites were then labeled with propidium iodide **(A)**, annexin V-FITC **(B)**, TUNEL kit **(C)** or MDC **(D)**. Heated promastigotes at 65°C for 15 min **(A)**, miltefosine (8 μM) **(B)**, DNAse (5 μg/mL) **(C)** and nutrient starvation (parasites growing for 3 days in PBS) **(D)** were used as positive controls. Fluorescence intensity was evaluated in a spectrofluorometer at 335/518 nm **(D)**, at 485/528 nm **(B,C)** or at 540/600 nm **(A)** of excitation/emission. Statistical analyses were made using One-way ANOVA followed by Tukey post-test to compare the untreated control group with the other treatments: ***p < 0.001, **p < 0.01 and ns (not significant).

The treatment with PF-429242 did not alter the permeability of the parasite plasma membrane as PI did not penetrate inside the treated promastigote (Figure 3A), similar to the untreated promastigotes and unlike the positive control of promastigotes heated at 65°C, which exhibited significant penetration of PI compared to the untreated control. Relative fluorescence units: ∼2,200 in control, ∼2,300 in cells treated with 3 μM PF-429242 and ∼2,700 after treatment with 6 μM PF-429242 (Figure 3A). These results show that the plasma membrane of promastigotes treated with PF-429242 is not disrupted.

Despite discussion regarding the occurrence of PS exposure in the external leaflet of the *Leishmania* plasma membrane, since there is a possibility of this phospholipid being absent or present in small amounts in this parasite membrane (Proto et al., 2013), the labeling of this phospholipid with annexin V-FITC has been widely used to detect an apoptosis-like process in *Leishmania* (Mesquita et al., 2013; Chouhan et al., 2015; Mallick et al., 2015; Bortoleti et al., 2018; Gonçalves et al., 2018; Tomiotto-Pellissier et al., 2018; Miranda-Sapla et al., 2019). In this work, this probe was used to evaluate the PS externalization in *L. infantum* promastigotes after treatment with PF-429242. The results showed that was no PS externalization in treated *L. infantum* promastigotes when compared with the untreated control, because the fluorescence intensity of the groups was similar (Figure 3B – Relative fluorescence units: ∼700 in control, ∼695 in cells treated with 3 μM PF-429242 and ∼940 after treatment with 6 μM PF-429242). Miltefosine was used as a positive control, which showed a significant increase (Relative fluorescence units ∼1,500) in annexin V-FITC staining (Figure 3B). In addition, DNA fragmentation was not observed in promastigotes treated with PF-429242 when compared with the untreated control, as the fluorescence intensity of the groups was similar (Figure 3C – Relative fluorescence units: ∼3,600 in control, ∼3,900 in cells treated with 3 μM PF-429242 and ∼3,800 after treatment with 6 μM PF-429242), as showed by TUNEL staining. DNAse (5 μg/mL) was used as a positive control and significantly increased the stain of cells with TUNEL (Figure 3C – Relative fluorescence units ∼5,800).

### PF-429242 Induces the Formation of Autophagic Vacuoles in *L. infantum* Promastigotes

The formation of autophagic vacuoles inside the parasites was evaluated by staining with MDC. After treatment, there was increased formation of autophagic vacuoles in *L. infantum* promastigotes, which can be seen through an increase in fluorescence intensity in the promastigotes treated with PF-429242 at 3 μM (Figure 3D). After treatment with PF-429242 at 6 μM there was no difference in the MDC fluorescence compared to the untreated control. Parasites growing for 3 days under malnutrition conditions (cultivated in PBS) were used as a positive control and in this group there was also an increase in autophagic vacuoles formation (Figure 3D – Relative fluorescence units: ∼140 in control, ∼440 in cells treated with 3 μM PF-429242, ∼280 after treatment with 6 μM PF-429242 and ∼480 in starvation group).

### PF-429242 Induces Flagellar Defects in *L. infantum* Promastigotes

**FIGURE 4:**
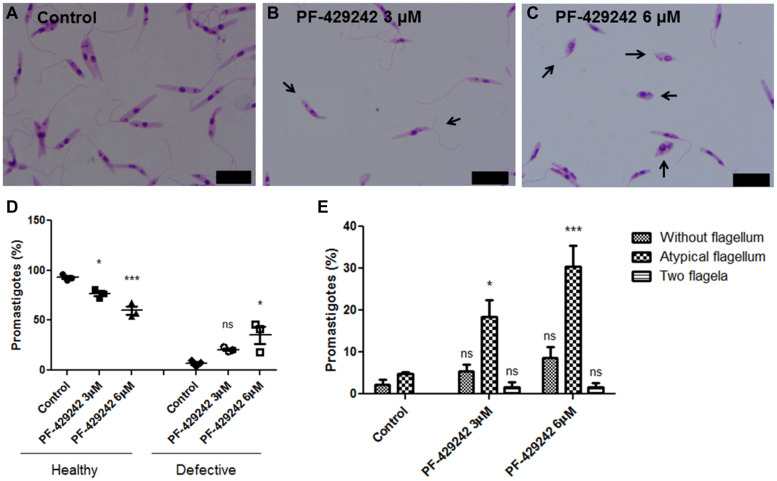
Morphological aspects of *L. infantum* promastigotes treated with PF-429242 observed by optical microscopy. The morphology of *L. infantum* promastigotes was determined after 72 h incubation with 3 and 6 μM PF-429242. The cells were washed with PBS, fixed with 4% paraformaldehyde and labeled with Giemsa. Slides were mounted and photographed on a fluorescence microscope (Olympus BX53). **(A)** Control; **(B)** PF-429242 at 3 μM; **(C)** PF-429242 at 6 μM. Black arrows indicate parasites with flagellar defects, such as without flagellum, atypical flagellum or two flagella. Bar = 10 μm. **(D)** Quantitative analysis of the percentage of healthy promastigotes and promastigotes with flagellar defects (defective) after treatment with PF-429242, when compared to the control. **(E)** Quantitative analysis of flagellar defects in untreated promastigotes and promastigotes treated with PF-429242 at 3 and 6 μM: without flagellum, atypical flagellum and two flagella. Statistical analyses were made using One-way ANOVA followed by Tukey post-test to compare the untreated control group with the other treatments: ***p < 0.001, *p < 0.05 and ns (not significant).

*Leishmania infantum* promastigotes treated or not with PF-429242 were fixed, Giemsa-stained and photographed (Figure 4). Treated parasites exhibited flagellar defects, such as promastigotes without flagellum, with atypical flagellum or parasites presenting two flagella (Figures 4B,C – black arrows). Quantitative analysis revealed a significant decrease in the percentage of promastigotes without flagellar defects (healthy) after treatment with PF-429242 (3 and 6 μM) and an increase in the percentage of promastigotes with flagellar defects (defective) after treatment with PF-429242 (6 μM), when compared to the control (Figure 4D). In control group, the percentage of promastigotes with flagellar defects was approximately 7%, while in promastigotes treated with PF-429242 at 3 μM was approximately 21% and at 6 μM was approximately 35% (Figure 4D). Furthermore, quantitative analysis of each of the flagellar defects was performed. Figure 4E shows that the most common and significant change after treatment was parasites with an atypical flagellum.

**FIGURE 5:**
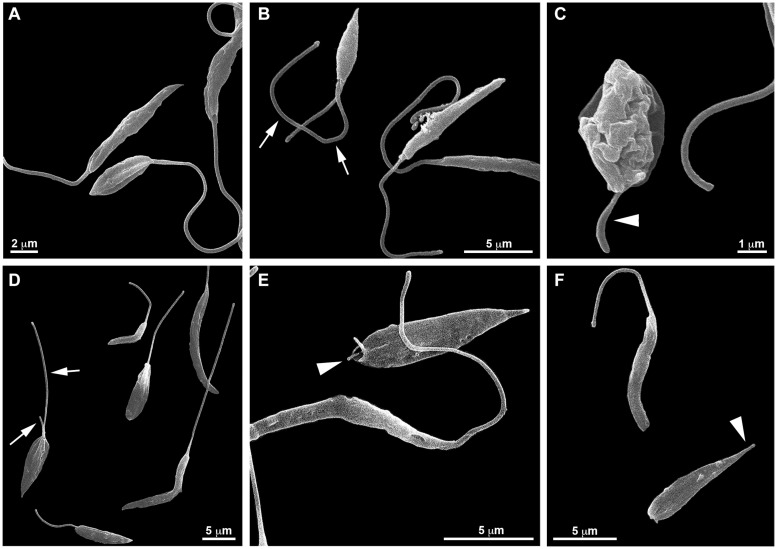
Effects of PF-429242 on *L. infantum* promastigote morphology using scanning electron microscopy (SEM). SEM micrographs of non-treated **(A)** and PF-429242-treated parasites for 72 h **(B–F)**. Non-treated parasites presented regular morphology; elliptical or elongated cellular bodies with very long flagellum. Parasites treated with 3 μM PF-429242 presented two flagella (arrow) **(B)** or an atypical flagellum (arrowhead) **(C)**. The same characteristics were observed with PF-429242 at 6 μM; promastigotes with two flagella (arrow) **(D)** and with atypical flagella (arrowhead) **(E,F)**.

SEM analyzes (Figure 5) also revealed that the treatment with PF-429242 induced flagellar defects in *L. infantum*. Promastigotes treated with PF-429242 (3 μM) presented two flagella (Figure 5B – arrow) and atypical flagella (Figure 5C – arrowhead). The same characteristics were observed after treatment with PF-429242 (6 μM), such as promastigotes with two flagella (Figure 5D – arrow) and with atypical flagella (Figures 5E,F – arrowhead). These data corroborate with the data obtained by optical microscopy.

### Promastigotes and Intracellular Amastigotes of *L. infantum* Presented Anormal Ultrastructure After Treatment With PF-429242

**FIGURE 6:**
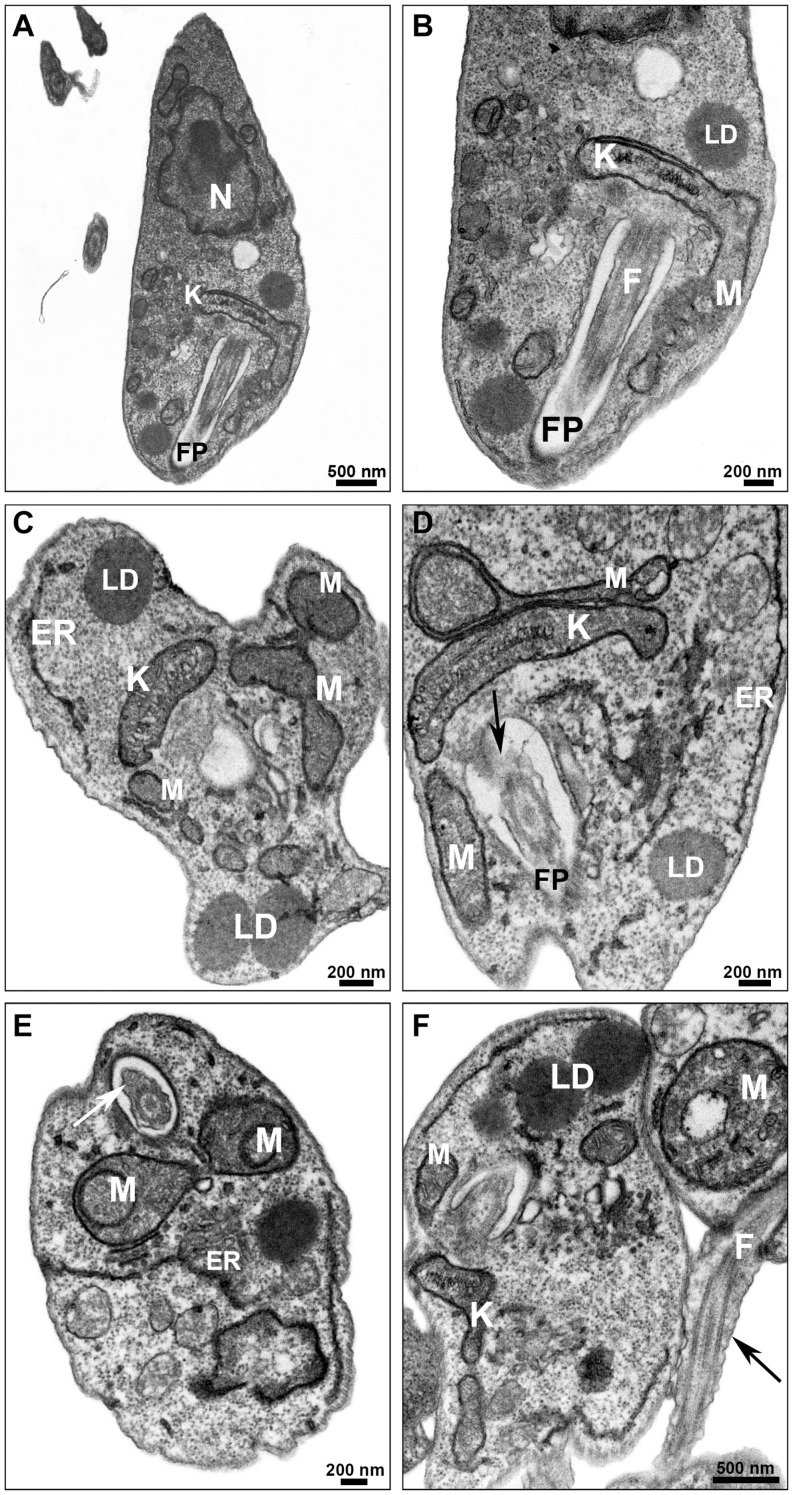
Ultrastructural effects in *L. infantum* promastigotes treated with PF-429242: mitochondrial damage and flagellar defects. Transmission electron micrographs of non-treated **(A,B)** and PF-429242-treated parasites **(C–F)**. Non-treated parasites presented normal morphology, with homogeneous cytoplasm **(A)** and well-preserved structures **(A,B)**. Parasites were incubated with 3 μM **(C,D)** and 6 μM **(E,F)** PF-429242 for 72 h. Cells treated with 3 μM presented atypical morphology **(C)**, alterations were seen in mitochondria morphology and cristae, as well as in the kinetoplast **(C,D)**. Flagellar membrane disruption (arrow) inside the flagellar pocket can be observed **(D)**. The similar damages were visualized in parasites treated with 6 μM; flagellar membrane enlargement (arrows) **(E,F)**, mitochondria alteration in morphology and cristae **(E,F)** and kinetoplast **(F)**. ER, endoplasmic reticulum; F, flagellum; FP, flagellar pocket; K, kinetoplast; LD, lipid droplets; M, mitochondria; N, nucleus.

**FIGURE 7:**
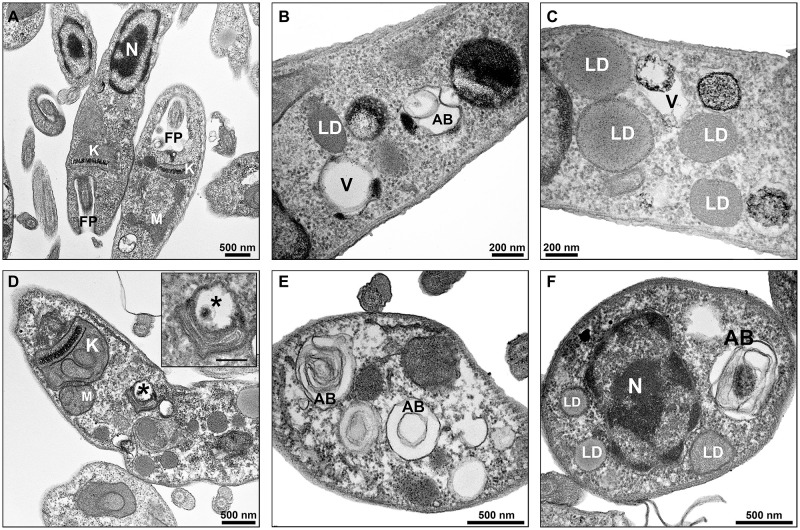
Ultrastructural effects in *L. infantum* promastigotes treated with PF-429242: autophagic bodies, lipid droplets and recruitment of endoplasmic reticulum. Transmission electron micrographs of non-treated **(A)** and PF-429242-treated parasites **(C–F)**. Non-treated parasites presented regular morphology, with homogeneous cytoplasm and well-preserved structures **(A)**. Parasites were incubated with 3 μM **(B,C)** and 6 μM **(D,F)** PF-429242 for 72 h. Note that cells treated with PF-429242 (3 μM) present some vacuoles (V) and autophagic bodies (AB) **(B)**. The number of lipid droplets (LD) increased after treatment **(C)**. In the figure **(D)**, recruitment of endoplasmic reticulum surrounding a vesicle (inset) can be observed, moreover a damaged kinetoplast (K) is seen **(D)**. Autophagic bodies are also visualized after treatment with PF-429242 at 6 μM **(D–F)**. AB, autophagic bodies FP, flagellar pocket; K, kinetoplast; LD, lipid droplets; M, mitochondria; N, nucleus; V, vacuole.

Promastigotes treated with PF-429242 at 3 μM presented atypical morphology (Figures 6, 7), alterations were seen in mitochondria morphology and cristae, as well as in the kinetoplast (Figures 6C,D). Flagellar membrane disruption (arrow) inside the flagellar pocket could be observed (Figure 6D). Similar damages were visualized in promastigotes treated with PF-429242 at 6 μM such as flagellar membrane enlargement (Figures 6E,F – arrows), and mitochondria alteration in morphology and cristae (Figures 6E,F) and kinetoplast (Figure 6F). In addition to mitochondrial and flagellar changes, promastigotes treated with PF-429242 (3 μM) presented vacuoles (V) and autophagic bodies (AB) (Figure 7B). The number of lipid droplets (LD) also increased after treatment (Figure 7C). In Figure 7D, it was possible to observe a recruitment of endoplasmic reticulum surrounding a vesicle (inset). Autophagic bodies were also visualized after treatment with PF-429242 at 6 μM (Figures 7E,F). It was also observed that the plasma membrane of treated promastigotes was not disrupted (Figures 6, 7). It is interesting to highlight that the changes observed in *L. infantum* promastigotes by TEM are in accordance with the changes previously reported in this work, such as mitochondrial changes, accumulation of neutral lipids, formation of autophagic vacuoles, the absence of rupture of the plasma membrane and the presence of flagellar defects.

**FIGURE 8:**
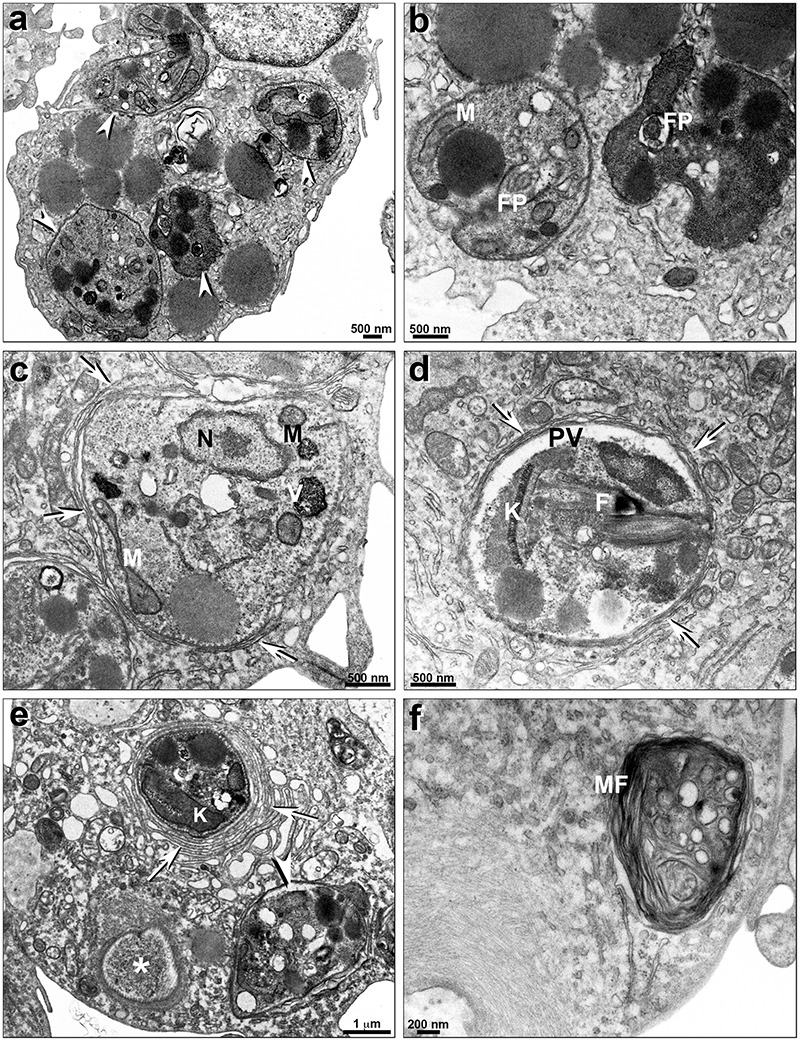
Ultrastructural effects of PF-429242 in *L. infantum* intracellular amastigotes. Transmission electron micrographs of non-treated **(a,b)** and PF-429242-treated peritoneal macrophages infected with parasites **(c–f)**. It is possible to see a number of intracellular parasites (arrowheads) in non-treated infected macrophage **(a)**. No parasite damages were seen in non-treated macrophages **(a,b)**. Cells were incubated *in vitro* with 14 μM **(c,d)** and 28 μM **(e,f)** PF-429242 for 72 h. Macrophages treated with 14 μM presented altered mitochondria **(c)** and endoplasmic reticulum profiles (arrows) surrounding the parasitophorous vacuole (PV) **(c,d)**. Moreover, a damaged amastigote is seen inside the PV **(d)**. Treatment with 28 μM provoked severe alterations in amastigotes **(e)**, a completely destroyed parasite inside PV (asterisks) and *Leishmania* plasma membrane retraction (arrowhead) **(e)**. Intense macrophage endoplasmic reticulum profiles surrounding the PV (arrows) can clearly be seen **(e)**. Myelinic figures, a classic ultrastructural autophagic characteristic, were seen after 28 μM treatment **(f)**. FP, flagellar pocket; N, nucleus; K, kinetoplast; M, mitochondria; MF, myelinic figures; PV, parasitophorous vacuole; V, vacuole.

*Leishmania infantum* intracellular amastigotes treated with PF-429242 at 14 μM presented altered parasite mitochondria (Figure 8c) and endoplasmic reticulum profiles (arrows) surrounding the parasitophorous vacuole (PV) (Figures 8c,d). Moreover, a damaged amastigote can be seen inside the PV (Figure 8d). Treatment with PF-429242 at 28 μM provoked severe alterations in amastigotes (Figure 8e), including a completely destroyed parasite inside the PV (asterisks) and *Leishmania* plasma membrane retraction (arrowhead) (Figure 8e). Intense macrophage endoplasmic reticulum profiles surrounding the PV (arrows) can clearly be seen and alterations in kinetoplast (Figure 8e). Myelinic figures, a classic ultrastructural autophagic characteristic, were seen after treatment with PF-429242 at 28 μM (Figure 8f). These results show that mitochondrial damage and autophagy are involved in the death of intracellular amastigotes of *L. infantum* as was observed in promastigotes.

### PF-429242 Stimulates NO and TNF Production by *L. infantum-*Infected Macrophages

**FIGURE 9:**
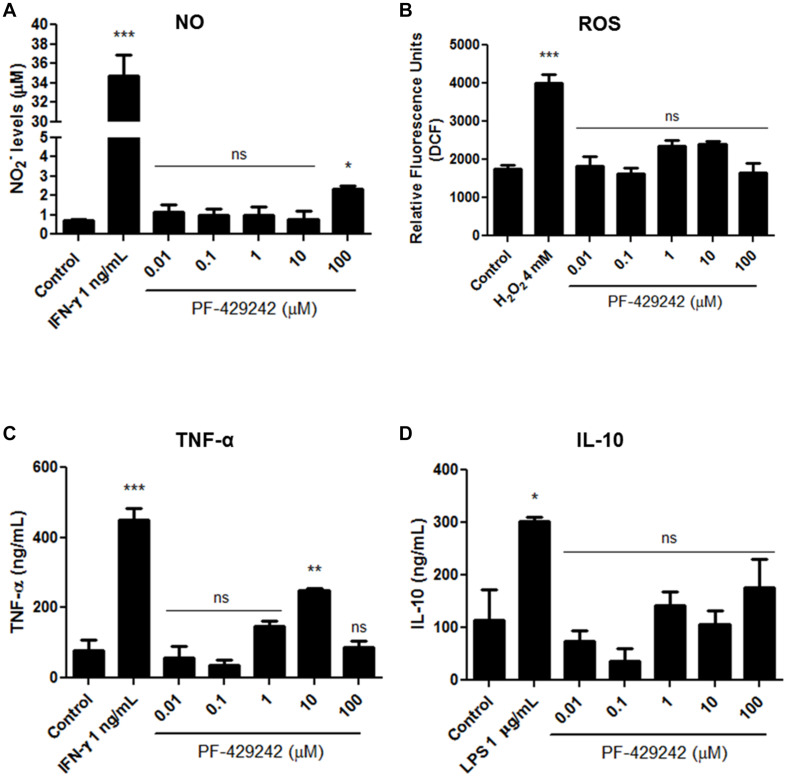
NO, ROS, TNF-α, and IL-10 levels produced by *L. infantum*-infected macrophages treated with PF-429242. **(A)** Supernatants from cultures of *L. infantum-*infected macrophages, treated or not with PF-429242, were collected and NO levels were determined by Griess method. Nitrite content was quantified by comparison with a sodium nitrite standard curve. The absorbance was measured at 540 nm using a spectrophotometer. IFN-γ (obtained from L1210 cell supernatant) at 1 ng/mL was used as a positive control. **(B)** Macrophages from BALB/c mice were infected with *L. infantum* promastigotes for 24 h at 37°C. PF-429242 was added and maintained for 72 h. After this time, H_2_DCFDA (20 μM) was added for 30 min. The read was made in a spectrofluorometer (485/528 nm). *L. amazonensis*-infected macrophages stimulated with H_2_O_2_ (4 mM) for 30 min were used as a positive control. **(C,D)** The supernatant of culture of macrophages infected with *L. infantum* amastigotes and treated with PF-429242 for 72 h was used. The TNF-α **(C)** and IL-10 **(D)** levels were detected by sandwich enzyme-linked immunosorbent assay (ELISA) ABTS protocol using the diagnostic kits. The absorbance was read on a spectrophotometer using 405 nm with wavelength correction set at 650 nm. Values of optical density were calculated from the standard curve for each cytokine. Supernatant from cells treated with IFN-γ (1 ng/mL) was used as a positive control for the production of TNF-α and treated with LPS (1 μg/mL) was used as a positive control for the production of IL-10. Statistical analysis by One-way ANOVA followed by Tukey post-test was performed to compare the control (infected with *L. infantum* and untreated) with the other treatments: ***p < 0.001, **p < 0.01, *p < 0.05 and ns (not significant).

NO, ROS, TNF-α, and IL-10 production by macrophages infected with *L. infantum* and treated with PF-429242 was also assessed. Interestingly, PF-429242 induced an increase of NO levels at 100 μM PF-429242 (increase of 240%) when compared with the control group (Figure 9A), but no increase in ROS levels was observed (Figure 9B). In addition, treatment with PF-429242 at 10 μM significantly induced TNF production (237%) when compared to the untreated infected macrophages (Figure 9C), while the IL-10 production was not altered in infected macrophages treated with the different concentrations of PF-429242 or left untreated (Figure 9D). IFN-γ, LPS and H_2_O_2_ were used as positive controls. IFN-γ (1 ng/mL) significantly induced the NO and TNF production in untreated infected macrophages (Figures 9A,C, respectively), LPS (1 μg/mL) significantly induced the IL-10 production (Figure 9D) and H_2_O_2_ significantly induced the ROS production (Figure 9B). These data show that PF-429242 induced the production of substances from macrophages (NO and TNF) that favor the development of an immune response conducive to *Leishmania* death, but did not induce the production of the regulatory cytokine, IL-10, at the concentrations evaluated.

## Discussion

The present study reports the antileishmanial effect of PF-429242, an inhibitor of serine proteases, against *L. infantum*, the causative agent of VL in Latin America (Burza et al., 2018). PF-429242 exhibited an effect against both the promastigote and intracellular amastigote forms of *L. infantum* and, interestingly, had low toxicity against macrophages. The SI (the ratio between the effect of the compound on macrophages and promastigotes or amastigotes) was calculated, as this is an important safety metric and shows if the compounds are selective for the parasites over the host cells. The possibility of a compound exerting toxic effects in the human host decreases with the increase of the SI (Ortega et al., 2019). PF-429242 was found to be 68 times more toxic to promastigotes (SI = 68) and 13 times more toxic to amastigotes (SI = 13) compared to the host cells, demonstrating good selectivity toward the parasites instead of the host cells. This fact is very important because one of the problems related to the current chemotherapy of leishmaniasis is the toxicity of the drugs available (Ghorbani and Farhoudi, 2018). Studies have already shown that serine protease inhibitor compounds may have a leishmanicidal effect but little has been shown for PF-429242 specifically. Souza-Silva et al. (2015) showed that an epoxy-α-lapachone inhibited serine protease-enriched fractions from *L. amazonensis*, was effective against promastigotes and amastigotes of this parasite and reduced paw lesions in experimentally infected BALB/c mice. Paik et al. (2014) tested the antileishmanial activity of a fraction rich in serine protease inhibitors obtained from a potato tuber. This fraction revealed a strong inhibitory effect against serine proteases of an *L. donovani* cell lysate and inhibited the growth of promastigotes (IC_50_ = 312.5 μg/mL) and amastigotes (IC_50_ = 82.3 μg/mL) of this parasite.

As PF-429242 demonstrated good results against *L. infantum*, the antileishmanial effect of this compound was further elucidated. Promastigotes stained with MitoTracker^®^ Red CM-H2XROS and rhodamine 123 revealed that this compound increased the ΔΨ_*m*_, while MitoSOX staining revealed an increase in the mitochondrial superoxide production. It is important to highlight that the maintenance of ΔΨ_*m*_ is vital for cell metabolic processes and consequently for cell survival (Fonseca-Silva et al., 2011); thus both increased (hyperpolarization) or decreased (depolarization) ΔΨm can have negative consequences for mitochondrial integrity (Dagnino et al., 2018). In addition, superoxide generation has also been related to mitochondrial dysfunction, being indicative of damage to this organelle (Lafargue et al., 2017). TEM analyzes revealed alterations in mitochondria morphology and cristae, as well as in the kinetoplast. Together, these data indicate that the mitochondrion is a potential target of PF-429242 in *L. infantum*. The mitochondria of trypanosomatids, such as *Leishmania*, has been shown to be a potential drug target for treatment, because it is a unique organelle and vital for this protozoan. Moreover, it has a peculiar structure with many differences from the mammalian mitochondria, which is highlighted by the presence of an enlarged region containing kDNA named the kinetoplast (Fidalgo and Gille, 2011).

In addition, it was shown that the treatment with PF-429242 increased the accumulation of neutral lipids in *L. infantum* promastigotes, as revealed by Nile Red staining and by TEM, in which an increase in the number of LDs, organelles rich in neutral lipids, mainly triacylglycerol and cholesterol esters, could be observed (Boren and Brindle, 2012). This fact may be related to the mitochondrial dysfunction, being a possible cellular defense mechanism, since with the mitochondria compromised the cell accumulates lipids as an energy source. Lee et al. (2013) already described this compensatory mechanism in which mitochondrial dysfunction induces accumulation of neutral lipids during stress conditions *in vitro* and *in vivo*. Aon et al. (2014) also reported that the lipid accumulation inside the cells can be seen as an adaptive response to supply the cell energy without affecting the mitochondrial and cellular redox status and in order to maintain a low concentration of lipotoxic intermediates. It has previously been reported that mitochondrial changes can cause the deposition of lipids in liver cells (Li et al., 2020). The association between mitochondrial dysfunction and accumulation of neutral lipids was also demonstrated in *Leishmania* after treatment with different compounds (Kathuria et al., 2014; Scariot et al., 2017).

Furthermore, PF-429242 treatment induced oxidative stress in *L. infantum* promastigotes. Hyperpolarization of ΔΨm is able to cause an increase of ROS production as was already observed during hyperglycemic conditions (Yu et al., 2006) and in *L. infantum* promastigotes treated with sugiol, a compound extracted from the bark of *Cupressus lusitanica* (Scariot et al., 2019). Thus, the hyperpolarization of ΔΨm could cause the increase in ROS production after treatment with PF-429242. In addition, it is important to note that *Leishmania* subtilisin is a maturase for the trypanothione reductase (TR) system, which works to detoxify ROS and consequently to maintain redox homeostasis (Swenerton et al., 2010). As PF-429242 is a subtilisin inhibitor, the correct functioning of the TR system may be compromised and the intracellular ROS accumulates, which also could explain the increase in ROS levels within the treated promastigotes.

Three types of cell death have been described in trypanosomatids: apoptosis-like, necrosis and autophagy. All these process are better described in Metazoans and currently it is known that the phenotypic characteristics of each process may differ between Metazoans and Trypanosomatids. Apoptosis is a process of regulated cell death essential for different biological processes, such as the removal of non-functional or damaged cells (Menna-Barreto, 2019). During this process there is a reduction in cell volume, DNA fragmentation, PS externalization and bleb formation in the plasma membrane with intact permeability. As most of the molecular machinery of unicellular organisms appears to differ from those of multicellular organisms (Kaczanowski et al., 2011), the term “apoptosis-like” is preferred when referring to the process in *Leishmania*, and this term has been adopted in this work. Necrosis is another cell death subtype that may occur in a regulated or unregulated manner (Proto et al., 2013), but plasma membrane disruption is the main feature of this event (Menna-Barreto, 2019). Autophagy is an important homeostatic mechanism in response to cellular stress, in which the cell degrades its constituents, such as old or unnecessary proteins and organelles, recycling them (Proto et al., 2013). In *Leishmania*, metacyclogenesis is dependent on autophagy. However, when exacerbated, the autophagy can cause cell death (Menna-Barreto, 2019). The occurrence of autophagy is characterized by massive autophagic vacuolization of the cell cytoplasm (Smirlis et al., 2010). Stress conditions, such as treatment with different drugs, can induce an autophagic phenotype in trypanosomatids, causing the formation of myelin-like structures, multivesicular bodies and an increase in autophagic vacuole number. In addition, endoplasmic reticulum profiles have also been observed during autophagy in trypanosomatids (Menna-Barreto, 2019).

In this work, the treatment with PF-429242 did not alter the permeability of the plasma membrane, did not induce PS externalization and did not cause DNA fragmentation in *L. infantum* promastigotes, suggesting that the cell death process may not be necrosis or apoptosis-like. On the other hand, an increase in the formation of autophagic vacuoles was observed in the treated promastigotes, which was observed by MDC staining and TEM. In addition, by TEM there was recruitment of endoplasmic reticulum. These results show that autophagy could be part of the mechanism of action for PF-429242 in *L. infantum* promastigotes. Silva-Lopez et al. (2007) already reported that a serine protease inhibitor obtained from the sea anemone *Stichodactyla helianthus* induced the formation of vesicles similar to autophagic vacuoles in the cytoplasm of *L. amazonensis* promastigotes.

It is interesting to note that PF-429242 induced morphological changes related mainly to the flagellar portion of *L. infantum* promastigotes, in which the presence of parasites without flagellum, with atypical flagellum or with two flagella were observed. These characteristics were observed by optical microscopy, SEM and TEM. Silva-Lopez et al. (2007) also showed changes in the flagellar pocket of *L. amazonensis* promastigotes treated with different serine proteases inhibitors. Swenerton et al. (2010) reported that subtilisin-deficient *L. donovani* amastigotes displayed a retained flagella. These facts indicate that the flagellar alterations observed in *L. infantum* promastigotes treated with PF-429242 could be related to subtilisin inhibition.

It has been reported that the autophagic process may be related to peculiar morphological changes, mainly in the size of the flagellum, mitochondrial alterations and ROS production (Menna-Barreto, 2019). Additionally, in tumoral cells, the induction of autophagy by ROS was identified, with it appearing that mitochondrial superoxide is the major species involved in the induction of this process (Bellot et al., 2013). The autophagy observed in *L. infantum* promastigotes treated with PF-429242 may also be related to the recycling of abnormal lipids and damaged organelles, such as mitochondria (Medina et al., 2012).

In addition to assessing the mechanism of action of PF-429242 in *L. infantum* promastigotes, the mechanism involved in the death of intracellular amastigotes treated with PF-429242 was also investigated. Intracellular amastigotes inside macrophages treated with PF-429242 presented altered mitochondria and the PV containing the parasite was surrounded by endoplasmic reticulum profiles. Myelinic figures, a classic ultrastructural autophagic characteristic, were also seen after treatment with PF-429242. These results show that mitochondrial damage and autophagy are involved in the death of intracellular amastigotes of *L. infantum*, as was observed in promastigotes.

Furthermore, PF-429242 treatment induced NO and TNF production by macrophages infected with *L. infantum* amastigotes but did not induce the production of a regulatory cytokine, IL-10. This shows that this compound is also able to modulate cellular responses, leading to the development of an immune response that is favorable to *Leishmania* death. The production of NO by macrophages infected with *L. infantum* and treated with PF-429242 can be explained, since treatment with this compound induces the death of amastigotes and with this occurs the reduction of the modulation of microbicidal functions of macrophages by *Leishmania*, consequently these host cells start to respond to the presence of the parasite, producing NO. Previously it was mentioned that a serine protease inhibitor induced NO production in infected macrophages with *L. donovani* (Paik et al., 2014).

## Conclusion

The serine protease inhibitor, PF-429242, exhibited good activity against *L. infantum* promastigotes and amastigotes, with selectivity for the parasite instead of the host cell. It was also shown that the parasite mitochondrion is a potential target of PF-429242. In addition, this compound increased the accumulation of neutral lipids, caused oxidative stress, induced autophagy and caused flagellar defects in *L. infantum* promastigotes. Mitochondrial damage and autophagy also can be involved in the death of *L. infantum* intracellular amastigotes after treatment with PF-429242. Furthermore, this compound modulated cellular responses stimulating NO and TNF production by *L. infantum-*infected macrophages. These data highlight the antileishmanial potential of the serine protease inhibitor PF-429242 with parasites displaying a number of cell changes that act together leading to their death.

## Data Availability Statement

The original contributions generated for this study are included in the article/supplementary material, further inquiries can be directed to the corresponding author/s.

## Ethics Statement

The animal study was reviewed and approved by Ethical Committee for Animal Handling - Universidade Federal do Rio de Janeiro, Fundação Oswaldo Cruz-Fiocruz and Universidade Federal de Juiz de Fora.

## Author Contributions

PM conceived the original idea, carried out the experiments, scientific discussion, and wrote the manuscript. PG and VM carried out the experiments and scientific discussion. EC supported the project, scientific discussion, and wrote the manuscript. HM conceived the original idea, supported the project, scientific discussion, and wrote the manuscript. All authors contributed to the article and approved the submitted version.

## Conflict of Interest

The authors declare that the research was conducted in the absence of any commercial or financial relationships that could be construed as a potential conflict of interest.
